# Genome-Wide Association Study of Fruit Traits Using 109 Germplasm Accessions of *Camellia oleifera*

**DOI:** 10.3390/biology15060483

**Published:** 2026-03-18

**Authors:** Weiwei Xie, Yuyun Yu, Yiqing Xie, Yu Li, Yong Huang, Wenjun Lin, Miao Yu, Haichao Hu, Shipin Chen, Zhizhen Li

**Affiliations:** 1 Fujian Academy of Forestry, Fuzhou 350012, China; weiweixie2020@163.com (W.X.); xyqing168@163.com (Y.X.); huangyfujian@163.com (Y.H.); myu0321@163.com (M.Y.); 2 College of Forestry, Fujian Agriculture and Forestry University, Fuzhou 350002, China; yuyunyu2020@126.com (Y.Y.); yulitrees@163.com (Y.L.); linwenjun@fafu.edu.cn (W.L.); huhaichao@fafu.edu.cn (H.H.)

**Keywords:** *Camellia oleifera*, fruit traits, genetic structure, GWAS, SNP

## Abstract

*Camellia oleifera* Abel, recognized as a woody oil-producing tree species, possesses considerable economic significance. To improve the breeding efficiency of *C. oleifera*, it is crucial to elucidate the genetic foundation underlying the mechanisms regulating fruit traits. In this study, a total of 6,252,197 high-quality single-nucleotide polymorphisms (SNPs) were identified from 109 germplasm accessions. Through genetic structure analysis, these accessions were categorized into two distinct populations. The average fixation index (Fst) was found to be 0.0153, indicating weak population differentiation. The genome-wide association analysis (GWAS) identified 157 significant loci. From these loci, 110 candidate genes were selected, which were associated with disease resistance, reproduction, development, and RNA biosynthesis. Twenty-three genes were involved in metabolic pathways, including genetic information-processing protein families, metabolic protein families, terpenoids, and polyketides. The identification of gene loci closely related to fruit traits not only provides genetic data for studying the molecular mechanisms of fruit traits but also offers new research avenues for molecular breeding of *C. oleifera*.

## Simple Summary

*Camellia oleifera* is an important woody oil tree species in China. Understanding the genetic basis of its fruit traits is crucial for breeding improved varieties. In this study, we analyzed 109 *C. oleifera* accessions and identified 157 genetic loci associated with ten fruit traits. We found 110 candidate genes involved in reproduction, development, and metabolism. These findings provide valuable molecular markers for future breeding programs and contribute to our understanding of fruit development in woody plants.

## 1. Introduction

*Camellia oleifera* Abel, native to China, is a species within the genus *Camellia* of the family Theaceae. It is primarily a shrub but can also grow into a medium-sized tree. The seeds of *C. oleifera* possess rich contents of edible oil, also known as tea oil, making it one of four major edible oil producing crops worldwide [1,2,3]. Tea oil is rich in monounsaturated and polyunsaturated fatty acids, with significant uses in regulating blood cholesterol. Moreover, the oil contains significant contents of bioactive substances such as vitamin E and flavonoids, making it highly nutritious [4,5,6]. With a growth cycle spanning over a hundred years, *C. oleifera* represents a highly successful perennial cash crop.

The plant shows abundant variations in fruit shape and color among different germplasms. The most prevalent fruit colors include red, yellow, and green, while the fruit shapes can be categorized as spherical, orange-like, peach-like, navel-like, olive-shaped, jujube-shaped, and chicken-heart-shaped [7]. The key fruit traits affecting the oil production include fruit weight, fruit height, fruit diameter, fruit peel thickness, fresh seed yield rate, dry seed yield rate, and dry kernel yield rate [8,9]. These fruit traits vary among varieties and are affected by environment variations [10,11,12,13]. The diversity of fruit traits serves as an important foundation for selective breeding. In-depth analysis of the genetic basis underlying the diversity of fruit traits in *C. oleifera* germplasm and the identification of gene loci regulating fruit traits play crucial roles in the development of new varieties and molecular-assisted breeding [14,15,16].

However, due to the long breeding cycle, complex ploidy, and large genome size of *C. oleifera*, research on the genes regulating its fruit traits is relatively slow [17,18,19]. Genome-wide association study (GWAS) is an effective and convenient method for studying traits in plants and animals. By performing association analysis between genotypic and phenotypic data, significant loci or candidate genes can be identified [20]. Jia et al. [21] sequenced 916 foxtail millet varieties and identified a total of 2.58 million single-nucleotide polymorphisms (SNPs). They also constructed a haplotype map of the foxtail millet genome using 0.8 million high-quality SNPs. Furthermore, they conducted a genome-wide association study on 47 agronomic traits of the 916 varieties under five different environmental conditions, revealing a total of 512 loci associated with these traits. Wang et al. [22] conducted GWAS on traits related to chlorophyll content using 529 rice germplasm resources and identified 46 significantly associated loci. They selected three of these associated loci and constructed an F_2_ segregating population for validation. Wang et al. [23] also performed a GWAS using 219 soybean germplasm resources and detected a total of 12 quantitative trait loci (QTLs) related to net photosynthetic rate, photosynthetic rate, and stomatal conductance, which were distributed across 12 chromosomes. Zhang et al. [24] conducted GWAS on 11 agronomic traits of 312 sand pear germplasm resources from China, South Korea, and Japan, and identified 37 loci associated with 8 fruit quality traits and 5 loci associated with 3 fruit phenological traits. Among these, a candidate gene, PbrSTONE, was functionally validated to be involved in the regulation of stone cell formation [25]. These studies provide a framework for exploring the regulatory mechanisms of fruit traits in *C. oleifera*.

*C. oleifera* harbors rich genetic diversity in its germplasm resources [26]. This diversity provides a crucial gene pool for the genetic improvement of fruit traits in *C. oleifera* [27]. The development of high-throughput sequencing technology and the emergence of the *C. oleifera* genome offer an opportunity to develop SNP molecular markers covering the entire genome of *C. oleifera* [28]. However, compared with other fruit trees and crops, research on the regulatory mechanisms of fruit traits in *C. oleifera* remains limited. This study conducts a genome-wide association analysis on *C. oleifera* fruits for the first time and explores the genetic structure and diversity of *C. oleifera* populations. The objectives are to identify genetic loci related to the regulation of fruit traits and reveal potential molecular markers, providing a solid theoretical basis for the genetic improvement and molecular breeding of *C. oleifera*.

## 2. Materials and Methods

### 2.1. Plant Materials and Phenotypic Evaluation

The 109 *C. oleifera* germplasm accessions were planted in the experimental field of National *C. oleifera* Germplasm Resource Bank at Shaxian Shuinan State-owned Forestry Farm from Fujian Province (Supplementary File S1). These accessions originated from four provinces: Fujian (78 accessions), Jiangxi (17 accessions), Hunan (8 accessions), and Guangxi (6 accessions), with each accession assigned a unique identifier.

Fruit phenotyping was conducted during the fruit maturation period (November 2021). For each of the 109 *C. oleifera* accessions, approximately 20 mature fruits were randomly collected from different positions of the tree canopy to ensure representative sampling. The weight of each *C. oleifera* fruit was measured using an electronic balance, and the length and diameter of the fruits were accurately measured using a vernier caliper. The peel thickness of each fruit was measured at the top, middle, and bottom with a vernier caliper, and the average value was calculated. The fruit shape index (FSI) was defined as the ratio of fruit length to fruit diameter. Microsoft Excel 2021 was used to calculate FSI and other statistical data.

After peeling, the number of seeds in each fruit was recorded. The following traits were measured for each individual fruit: single fruit weight (g), fruit height (mm), fruit diameter (mm), fruit shape index, pericarp thickness (mm), seed number per fruit, fresh seed weight (g), and fresh seed yield rate. The weight of the shelled seeds was measured, and the average value was calculated to determine the fresh seed yield rate. Subsequently, the shelled seeds were dried in an oven and then weighed again to calculate the dry seed yield rate. The dried seeds were then shelled, and the weight of the kernels was measured to calculate the dry kernel yield rate. Based on the FSI, different categories of *C. oleifera* fruit shapes were distinguished, identified, and recorded.

Repeatability and broad-sense heritability (*H*^2^) for each trait were estimated using variance components from a linear mixed model implemented in the lme4 package (version 1.1.38) in R, with accession as a random effect. Repeatability was calculated as $ICC=\frac{V_{g}}{V_{g}+V_{e}}$. Broad-sense heritability was calculated as H2=VgVg+Vek, where k is the harmonic mean of the number of fruits per accession.

Correlation analysis among the ten fruit traits was performed using Pearson’s correlation coefficients based on the mean phenotypic values of the 109 accessions.

Phenotype aggregation for GWAS: The final phenotypic value used for GWAS was the arithmetic mean across all available fruits per accession. The number of fruits measured for each accession is provided in Supplementary File S2.

### 2.2. DNA Preparation and Sequencing

Genomic DNA was extracted from the leaves using a modified cetyltrimethylammonium bromide (CTAB) method [29]. After quantification by 1% agarose gel electrophoresis, the working DNA solutions were diluted to 100 ng/µL and stored at −20 °C. Subsequently, we constructed sequencing libraries for the 109 DNA samples using the ddRAD-seq method [30]. When the monoclonal detection results met the required standards, we performed 150 bp paired-end sequencing on each constructed sequencing library using the HiSeq 2500 platform. Based on the reference genome size of 2.95 Gb and the characteristics of our ddRAD protocol (SacI/MseI digestion, 300–500 bp fragment selection), we estimate that the libraries cover approximately 5.2% of the genome. The average sequencing depth in the captured regions was calculated as 7.3× per sample, which is sufficient for reliable SNP calling.

### 2.3. SNP Calling and Annotation

The paired-end raw data from the simplified genome sequencing were filtered using the software fastp (version 0.20.0) [31], with the parameters set as --adapter_sequence R1_adapter and --adapter_sequence_r2 R2_adapter to obtain paired-end clean data for each sample. To facilitate rapid searching and positioning during subsequent variant detection, the index of the *C. oleifera* genome was constructed using three software tools: SAMtools (version 1.16.1) [32], BWA (version 0.7.17) [33], and Picard (http://broadinstitute.github.io/picard/ accessed on 15 March 2026). The data were aligned to the genome using the mem algorithm of BWA, and the results were then sorted using the sort function of SAMtools to obtain BAM files of various qualities. Subsequently, the alignment rate was calculated using SAMtools. PCR duplicates were removed from the BAM files using the MarkDuplicates tool of Picard, ultimately generating BAM files of various qualities containing the aligned results. Next, variant detection was performed on the BAM files of various qualities by chromosome using the HaplotypeCaller module of the Genome Analysis Toolkit (GATK) (version 4.3.0.0) [34], generating corresponding gVCF files.

In the second step, the gVCF files of different germplasm resources were merged by chromosome using the CombineGVCFs module of GATK. Then, population variant detection was performed on the merged gVCF files using the GenotypeGVCFs module of GATK, obtaining VCF files for each chromosome. Finally, the VCF files for specific chromosomes were merged into a single genome-wide VCF file using the MergeVcfs tool of GATK.

In the third step, single-nucleotide polymorphisms (SNPs) and insertions/deletions (INDELs) were extracted using the SelectVariants tool of GATK, and then filtered and flagged using the VariantFiltration tool of GATK (SNP filtering criteria: QD < 2.0||MQ < 40.0||FS > 60.0||SOR > 3.0||MQRankSum < −12.5||ReadPosRankSum < −8.0; INDEL filtering criteria: QD < 2.0||FS > 200.0||SOR > 10.0||MQRankSum < −12.5||ReadPosRankSum < −8.0). Finally, the filtered SNPs and INDELs were extracted again using the SelectVariants tool of GATK, obtaining the final VCF files for SNPs and INDELs.

The identified SNPs were annotated using the ANNOVAR (version 2020Jun08) [35] software. First, a database was constructed using the FASTA file and GFF3 annotation file of the *C. oleifera* genome. Then, the obtained VCF files for SNPs were annotated.

### 2.4. Genetic Structure Analysis

Prior to population structure analysis, the SNP data were filtered using the Plink (version 1.9) [36] software based on criteria of missing rate (Geno 0.1), minor allele frequency (MAF 0.01), and linkage disequilibrium (LD) filtering (using the parameter --indep—pairwise 50 10 0.2). The filtered VCF files were then analyzed.

Phylogenetic analysis was performed using the filtered SNP dataset (274,489 high-quality SNPs after LD pruning). The VCF files were sorted and converted into PHYLIP format using the SortGenotypeFilePlugin module of TASSEL (https://tassel.bitbucket.io/). A phylogenetic tree was constructed using FastTree software (version 2.1.11) [37] with the approximate maximum likelihood method. The generalized time-reversible (GTR) model was selected as the nucleotide substitution model, as it is the most general and commonly used model for phylogenetic inference [38], accounting for different rates of transitions and transversions as well as unequal base frequencies. No multiple sequence alignment was required as the input consisted of SNP genotypes rather than sequences; the tree was built directly from the SNP data. Branch support was estimated using the Shimodaira-Hasegawa (SH)-like test implemented in FastTree with 1000 resamples. The resulting tree was visualized and annotated using FigTree (version 1.4.4, http://tree.bio.ed.ac.uk/software/figtree/, accessed on 15 March 2026).

Principal component analysis (PCA) was performed on the experimental *C. oleifera* population materials based on the detected SNPs using the Plink software. Following this, the vectors for each principal component were calculated using R software (version 4.2.2) [39], and the PCA scatter plot was plotted using R.

Additionally, the input file format was adjusted using Plink software to be compatible with the Admixture software (version 1.3.0) [40]. The files were then uploaded into the Admixture software, and the number of subpopulations (k value) was set from 1 to 10. The most appropriate number of subpopulations was determined based on the cross-validation error (CV error) values generated during the computation, and a population genetic structure matrix was generated. This matrix consists of the genetic component coefficients for each individual in each subpopulation.

Finally, charts were created using the pophelper (version 2.3.1) [41] and ggplot2 packages (version 3.4.0) in R to visualize the results of the population genetic structure matrix. The nucleotide diversity index (π), population differentiation index (Fst), and Tajima’s D values, which were calculated using the VCFtools software (version 0.1.16), were also visualized. Data were extracted from the computation result files, and the charts were plotted using the CMplot package in R.

### 2.5. GWAS Analysis

Prior to association analysis, SNP imputation was performed on the *C. oleifera* population using Beagle-5.5 [42]. The imputed data were subsequently filtered with Plink to retain markers with a minor allele frequency > 0.05 and a missing rate < 0.1, yielding a final set of high-quality SNPs for downstream analysis.

Genome-wide association analysis for the 10 fruit traits was conducted using the mixed linear model (MLM) implemented in EMMAX (Efficient Mixed-Model Association eXpedited). Given the weak population differentiation observed in our *C. oleifera* germplasm (mean Fst = 0.0153), we employed a kinship matrix (K) to account for genetic relatedness among individuals without including additional covariates such as principal components (PCs) or a Q matrix.

Specifically, we first calculated a pairwise identity-by-state (IBS) kinship matrix to estimate the genetic relationships among all 109 accessions. This kinship matrix was then incorporated as a random effect in the MLM:

(1) y=Xβ+Zμ+e

where:

y is the vector of phenotypic values for each trait;

β is the vector of fixed effects (including the SNP effect);

μ is the vector of random polygenic effects, with $Var\left(u\right)=\sigma_{\mu }^{2}K$;

e is the vector of residuals;

X and Z are incidence matrices for fixed and random effects, respectively.

The analysis was performed using EMMAX with default parameters, which first estimates the variance components under the null hypothesis (no SNP effect) and then tests each SNP individually while accounting for the kinship matrix. This approach has been shown to effectively control for both population structure and cryptic relatedness [43].

The genome-wide significance threshold was determined using the Bonferroni correction based on the total number of SNPs used in the GWAS (N = 2,812,326). Two thresholds were established:

Suggestive significance threshold: $P=1/N=3.56\times 10^{-7}$, corresponding to $-\log_{10}\left(P\right)=6.45$. This threshold indicates suggestive associations that warrant further investigation.

Highly significant (genome-wide) threshold: $P=0.05/N=1.78\times 10^{-8}$, corresponding to $-\log_{10}\left(P\right)=7.75$. This stringent threshold represents genome-wide significant associations after multiple testing correction.

The results were visualized using the R package CMplot, generating both Q–Q plots and Manhattan plots with suggestive (blue line) and genome-wide significant (red line) thresholds indicated.

### 2.6. Candidate Gene Screening

To determine an appropriate window size for candidate gene identification, we first analyzed the genome-wide LD decay pattern using the same set of 2,812,326 high-quality SNPs employed for GWAS. Based on the LD half-decay distance identified from this analysis, we defined the flanking region corresponding to significantly associated SNP markers as ±50 kb (50 kb upstream and 50 kb downstream of the significantly associated SNP marker). This window size ensures that genes in meaningful LD ($r^{2}\geq 0.1$) with the significant SNPs are captured while minimizing false positives.

Based on the annotation and functional annotation of SNP loci in *C. oleifera*, we considered the genes located within these loci as candidate genes. If a locus was simultaneously upstream and downstream of other genes, those upstream and downstream genes were also considered as candidate genes. If a locus was in an intergenic region, the nearest upstream and downstream genes were considered as candidate genes.

Candidate genes were annotated using GhostKOALA on the KEGG official website (https://www.kegg.jp/ghostkoala/, accessed on 15 March 2026) to obtain the relevant KO numbers (KEGG Orthology identifiers). Then, a Python (version 3.9.9) script was used for conversion to obtain pathway information at different levels. The Swiss-Prot database was downloaded from the NCBI official website (https://www.ncbi.nlm.nih.gov), and the protein sequences were compared to this database using BLAST (version 2.13.0) (BLAST: Basic Local Alignment Search Tool) to obtain the corresponding GO numbers. Subsequently, further analysis was conducted using Tbtools (version 1.120).

## 3. Results

### 3.1. SNP Variation Detection

**Figure 1:**
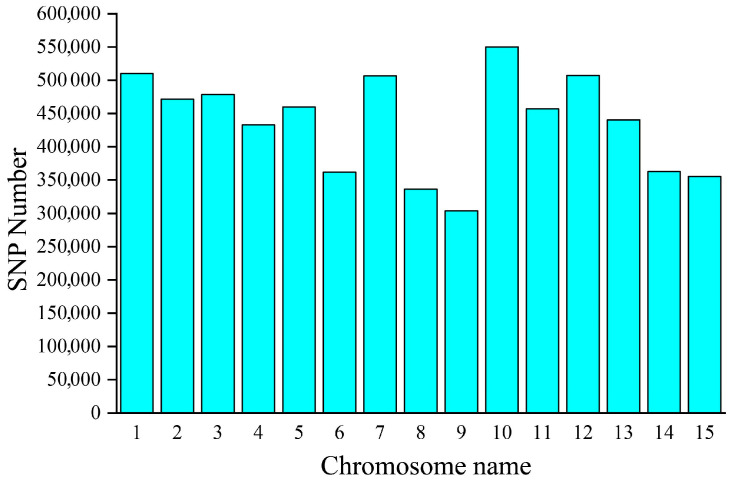
Distribution of SNPs on chromosomes.

After constructing libraries for each sample, approximately 126.7 G of raw data were obtained through paired-end sequencing. Following filtering using the fastp software, approximately 122.7 G of clean data were generated. Sequencing stats in Supplementary File S5. Variants were called from the aligned sequence files of the 109 *C. oleifera* samples using GATK. After initial filtering, a total of 6,535,538 raw population SNPs were obtained, distributed across all 15 chromosomes. Chromosome 10 harbored the highest number of SNPs (549,986), accounting for 8.42% of the total, while chromosome 9 contained the fewest (304,057 SNPs, 4.65%). The SNP counts for the remaining chromosomes were as follows, in descending order: Chr01 (510,385; 7.81%), Chr12 (507,496; 7.77%), Chr07 (506,689; 7.75%), Chr03 (478,721; 7.32%), Chr02 (471,624; 7.22%), Chr05 (459,979; 7.04%), Chr11 (456,825; 6.99%), Chr13 (440,464; 6.74%), Chr04 (432,971; 6.62%), Chr14 (362,843; 5.55%), Chr06 (361,753; 5.54%), Chr15 (355,498; 5.44%), and Chr08 (336,247; 5.14%). Overall, the number of markers on each chromosome exceeded 300,000, indicating a relatively uniform distribution across the genome (Figure 1).

Based on the reference genome size of 2.95 Gb and the characteristics of our ddRAD protocol (SacI/MseI digestion with 300–500 bp fragment selection), we estimate that the sequenced libraries cover approximately 5.2% of the genome. The average sequencing depth in the captured regions was calculated as 7.3× per sample (based on the average clean data of 1.126 Gb per accession and the estimated genome coverage), which is sufficient for reliable SNP calling as demonstrated by the identification of 6,252,197 high-quality SNPs.

Further annotation was performed on the variant sites located within exon regions. The results showed that 152,181 SNPs were situated in exons. Among these, 90,876 SNPs led to non-synonymous mutations, 56,530 resulted in synonymous mutations, 4562 were nonsense mutations, and 213 caused stop-loss mutations. These variants may have substantial effects on the traits of *C. oleifera*.

### 3.2. Genetic Structure

**Figure 2:**
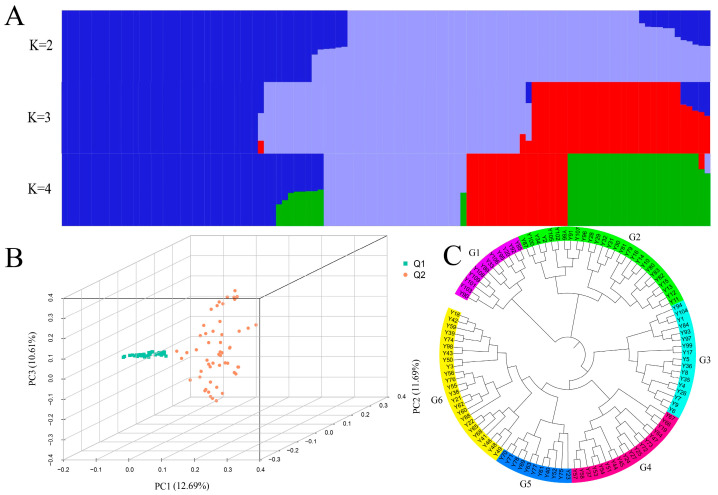
Population genetic analysis of 109 *C. oleifera* germplasm resources. Notes: (**A**) Population structure analysis using a stacked bar plot (K = 2–4), dark blue and light blue represent subpopulations Q1 and Q2, respectively. For K = 3 and K = 4, additional colors indicate further population substructure; (**B**) Principal component analysis (PCA); (**C**) Phylogenetic tree (based on the approximate maximum likelihood method and the generalized time-reversible model).

After filtering the missing rates, minor allele frequencies, and linkage disequilibrium (LD), 274,489 high-quality single-nucleotide polymorphism (SNP) loci were retained. The population structures of the 109 *C. oleifera* germplasm accessions were analyzed using the ADMIXTURE software (version 1.3.0). The number of subpopulations (k) was set from 1 to 10, and the cross-validation (CV) error values were calculated for each k. The results showed that the CV error was the lowest when k = 2, and it gradually increased as the value of k exceeded 2, indicating that dividing the germplasm resources into 2 groups at the genomic level was the most appropriate. The Q values for each germplasm accession were calculated for k values ranging from 2 to 10, and each accession was assigned to the subpopulation where its Q value was maximal (Figure 2A). The results indicated that when k = 2, the 109 *C. oleifera* germplasm accessions exhibited distinct structural blocks, suggesting effective grouping. From statistical analysis, it was found that subpopulation Q1 (dark blue) contained 48 accessions, while subpopulation Q2 (light blue) contained 61 accessions. There was evidence of gene flow and mutual gene infiltration between the two subpopulations. Further statistical analysis showed that the germplasm accessions in Q1 largely coincided with those in subgroups G1, G2, and G3 of the phylogenetic tree. Similarly, Q2 corresponded to subgroups G4, G5, and G6. Therefore, the division of 109 germplasm resources into 2 groups yielded effective results.

Principal component analysis (PCA) was performed on the SNP data using Plink software. The first three principal components, PC1, PC2, and PC3, collectively explained 34.99% of the variance, with PC1 accounting for 12.69%, PC2 for 11.69%, and PC3 for 10.61% (Figure 2B). The results were visualized using R, employing the two subpopulations inferred by ADMIXTURE for grouping, The first three principal components, which were highly representative of the variation, were selected for plotting.

A phylogenetic tree for 109 *C. oleifera* germplasm resources was constructed using the approximate maximum likelihood method in FastTree software (Figure 2C). The results indicate that the 109 *C. oleifera* germplasm resources can be divided into six subgroups: G1 to G6. Specifically, subgroup G1 contains 12 germplasm accessions, G2 has 27, G3 has 17, G4 has 18, G5 has 11, and G6 has 24. In subgroup G1, 2 accessions are from Fujian, 4 are from Hunan, and 6 are from Jiangxi. In subgroup G2, 14 accessions are from Fujian, 5 belong to Guangxi, 3 are from Hunan, and 5 are from Jiangxi. Subgroup G3 includes 11 accessions from Fujian and 6 from Jiangxi. Subgroups G4 and G5 contain 18 and 11 accessions, respectively, all from Fujian. In subgroup G6, apart from 1 accession from Hunan and 1 from Jiangxi, the remaining 22 are from Fujian. Further analysis reveals that the germplasm from Fujian in subgroups G4, G5, and G6 includes ‘Min 43’, ‘Min 46’, ‘Min 49’, and ‘Min 50’, as well as their hybrid offspring ‘Min Zayou’. These results suggest that the distinct geographical distribution characteristics of subgroups G4, G5, and G6 may be influenced by hybridization, leading to complex genetic backgrounds within these subgroups and subsequent population differentiation. Overall, there is no obvious geographical distribution pattern among the different subgroups of the 109 *C. oleifera* germplasm resources.

**Figure 3:**
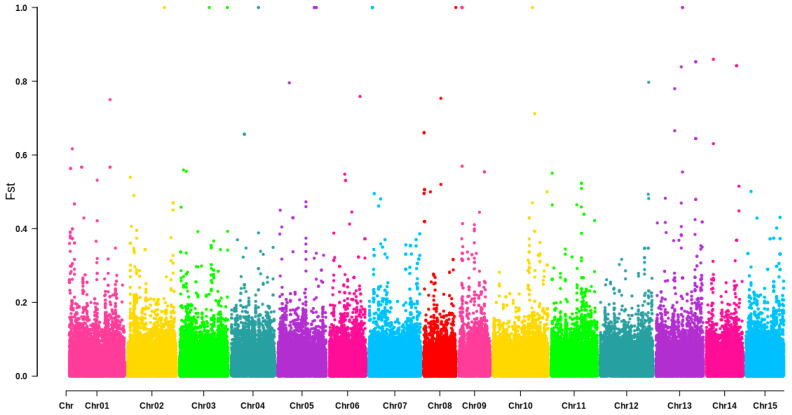
Distribution of Fst values on chromosomes of 109 *C. oleifera* germplasm resources.

Further calculations were performed to determine the distribution of Fst for each SNP marker across the whole genome (Figure 3). The Fst values for most SNP markers were below 0.2, and the average Fst value for all SNP markers across the genome was 0.0153. This suggests that the degree of differentiation between the Q1 and Q2 subpopulations is relatively weak, indicating weak genetic differentiation.

**Figure 4:**
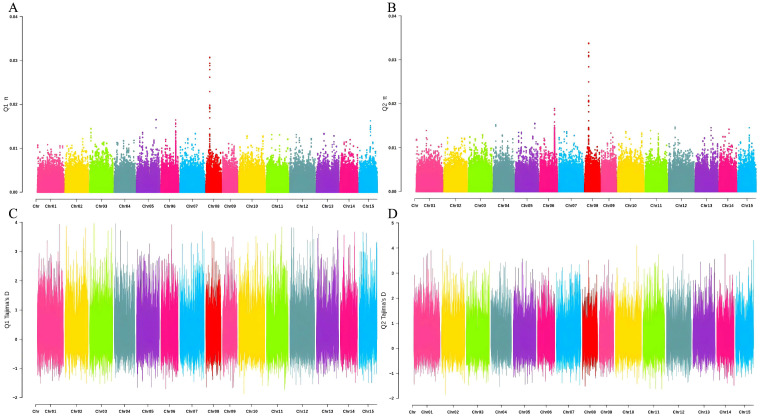
Distribution of π and Tajima’s D values across chromosomes. (**A**) π for Q1; (**B**) π for Q2; (**C**) Tajima’s D for Q1; (**D**) Tajima’s D for Q2.

The mean nucleotide diversity (π) values were $4.45\times 10^{-4}$ for subpopulation Q1 and $5.35\times 10^{-4}$ for subpopulation Q2, indicating comparable levels of genetic diversity between the two groups. Both subpopulations exhibited higher π values on chromosomes 6 and 8 (Figure 4A,B), suggesting that these genomic regions harbor well-preserved genetic variation. The π values for both subpopulations were mostly below 0.01, indicating relatively low genetic diversity in the *C. oleifera* germplasm, which may be attributed to long-term domestication.

Finally, the Tajima’s D values were calculated for the two subpopulations (Figure 4C,D). Tajima’s D is an indicator of selection within populations and is used to identify sequences that deviate from the neutral theory model by assessing the balance between mutation and genetic drift. The mean Tajima’s D value for the Q1 subpopulation across all SNP loci was 0.292, while it is 0.464 for the Q2 population. These positive Tajima’s D values suggested that the presence of rare alleles at low frequencies in both subpopulations, and there was a relatively high frequency of intermediate alleles. This may be due to the influence of domestication on the *C. oleifera* germplasm, leading to similar environments and uniform selection pressures within the populations.

### 3.3. Repeatability, Heritability, and Correlation Analysis of Fruit Traits

Repeatability and broad-sense heritability (*H*^2^) for the ten fruit traits were estimated based on approximately 20 fruits per accession (Supplementary File S4). Heritability estimates ranged from 0.614 to 0.957, with fruit shape index (*H*^2^ = 0.944) and pericarp thickness (*H*^2^ = 0.957) showing the highest values, indicating strong genetic control. Fruit diameter exhibited the lowest heritability (*H*^2^ = 0.614), suggesting greater environmental influence. Repeatability (ICC) values ranged from 0.126 to 0.666, consistent with heritability estimates.

Pearson’s correlation analysis revealed significant correlations among several fruit traits (Supplementary Figure S1). Strong positive correlations were observed between fruit weight and fruit diameter (r = 0.83, *p* < 0.001), as well as between fruit weight and fresh seed weight (r = 0.86, *p* < 0.001). Pericarp thickness showed strong negative correlations with fresh seed yield rate (r = −0.77, *p* < 0.001) and dry seed yield rate (r = −0.62, *p* < 0.001). These correlations suggest that some fruit traits may be controlled by common genetic factors or linked loci.

### 3.4. GWAS of Fruit Traits

Based on the LD decay analysis described in Section 2.6, which established an LD half-decay distance of 64 kb (Supplementary Figure S2), a total of 2,812,326 high-quality SNP markers spanning the *C. oleifera* genome were retained for GWAS analysis. Phenotypic data for 10 fruit traits from 109 germplasms are provided in Supplementary File S2.

**Figure 5:**
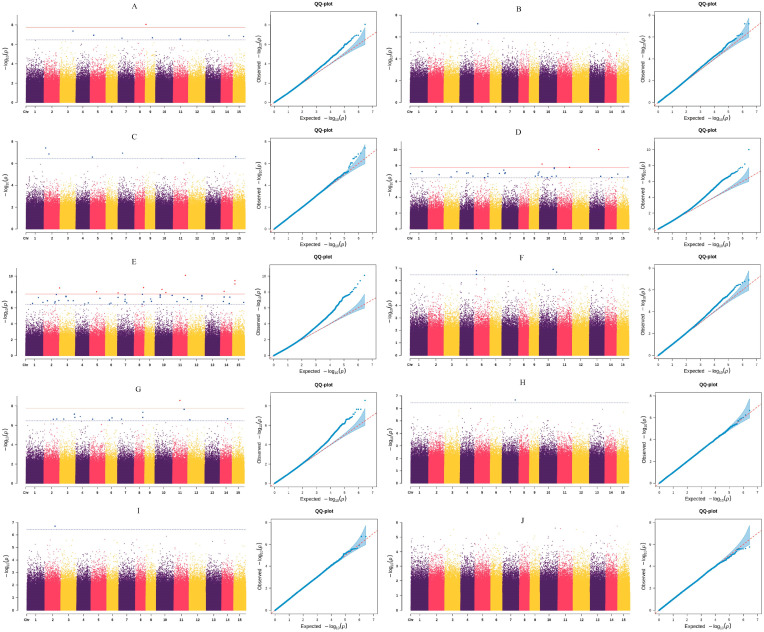
Manhattan plots (left) and Q-Q plot (right) for GWAS of 10 fruit traits in 109 *C. oleifera* germplasm resources. Notes: (**A**) Single fruit weight; (**B**) Fruit height; (**C**) Fruit diameter; (**D**) Fruit shape index; (**E**) Peel thickness; (**F**) Number of seeds per fruit; (**G**) Fresh seed weight; (**H**) Fresh seed yield; (**I**) Dried seed yield; (**J**) Dry benevolence yield. Blue and red dashed lines indicate suggestive (−log_10_*P* = 6.45) and genome-wide significant (−log_10_*P* = 7.75) thresholds, respectively. Each point represents a SNP.

The analysis was performed using the EMMAX mixed linear model, with significance thresholds set at $-\log_{10}\left(P\right)=6.45$ (suggestive) and $-\log_{10}\left(P\right)=7.75$ (genome-wide significant) for screening significant loci (Figure 5). The Q-Q plots for all ten traits showed no evidence of inflation (Figure 5), with genomic inflation factors (λ) estimated to be between 1.00 and 1.02. This confirms that population stratification was effectively controlled using only the kinship matrix in the EMMAX model.

All traits except for fruit height were found to be associated with significant loci. In total, 157 significant loci were identified (Figure 5; Supplementary File S3).

Specifically, nine SNP loci were significantly associated with single fruit weight, among which the locus at Chr09: 7869152 showed a highly significant association ($-\log_{10}\left(P\right)=8.05$). Two loci associated with fruit diameter were located at positions 43866733 and 43866738 on chromosome 5. For the fruit shape index, six significant loci were detected, with the most significant at Chr02: 20212286 ($-\log_{10}\left(P\right)=7.42$). Forty loci were associated with peel thickness; 37 showed significant and three showed highly significant associations. The SNP at Chr13: 111073692 was the most significantly associated ($-\log_{10}\left(P\right)=10.01$), followed by Chr10: 33172723 (8.17) and Chr11: 149010807 (7.76). Among the ten traits, the number of seeds per fruit had the highest number of associated loci (69 in total: 59 significant, 10 highly significant). The top three loci were Chr11: 153463775 ($-\log_{10}\left(P\right)=10.09$), Chr15: 29597100 (9.42), and Chr15: 29597154 (9.01). Five loci were associated with fresh seed weight, with the most significant at position 169280653 on chromosome 10 ($-\log_{10}\left(P\right)=6.91$). Twenty-three loci were associated with the fresh seed yield rate, with only one (Chr11: 87995439) being highly significant ($-\log_{10}\left(P\right)=8.56$). Only one locus (Chr07: 160272823) was associated with the dry seed yield rate ($-\log_{10}\left(P\right)=6.68$). Finally, two loci (Chr02: 124313904 and Chr02: 124313920) were associated with the dry kernel yield rate, both with a $-\log_{10}\left(P\right)$ value of 6.72 (Supplementary File S3).

### 3.5. Candidate Genes

Annotations were performed on the 157 significant SNP loci to identify for candidate genes. After removing duplicate genes, 110 candidate genes were identified (Supplementary File S3).

KEGG annotation of these 110 candidate genes was performed using the KEGG database (Table 1). The KEGG annotations indicated that the candidate genes are involved in various biological pathways, including genetic information processing, metabolic pathways, and terpenoid/polyketide metabolism (Table 1).

Gene Ontology (GO) annotation was performed for the candidate genes using BLAST against the Swiss-Prot database. Among the 110 candidate genes, 46 were successfully annotated with GO terms (Table 2). These 46 genes were assigned to the three main GO categories: molecular function (MF), cellular component (CC), and biological process (BP).

Within the MF category, six genes were annotated with functions such as heterocyclic compound binding, nucleotide binding, and disease resistance-related functions. In the CC category, nine genes were associated with terms including bounding membrane of organelle, protein-containing complex, and endoplasmic reticulum subcompartment. The BP category contained the largest number of genes (31), with annotations predominantly related to reproduction, development, RNA biosynthetic process, and defense response (Table 2). Notably, 16 out of the 31 BP-related genes were involved in reproduction, highlighting their potential roles in *C. oleifera* fruit development.

## 4. Discussion

In recent years, SNP molecular markers have assumed a critical role in elucidating biological genetic variation, interspecies relationships, and genetic breeding, owing to their numerous advantages in comparison to traditional breeding methods. These advantages include their abundance, wide distribution, ease of automated detection, low mutation rate, and rapid screening [44,45]. Currently, Simple Sequence Repeat (SSR) [46], Inter Simple Sequence Repeat (ISSR) [47], and Sequence-Related Amplified Polymorphism (SRAP) [48] molecular markers are commonly used to study the genetic diversity of *C. oleifera*. However, these markers have limitations, such as high time costs, low throughput, and relatively low accuracy, making it difficult to screen for highly polymorphic markers. Liu et al. [49] utilized SLAF-seq technology to sequence nine *C. oleifera* samples and successfully developed SNP markers. However, we did not use the *C. oleifera* genome as a reference for variant detection; consequently, marker development requires improvement and is insufficient for gene mapping of *C. oleifera* traits. Furthermore, the population size used in the study was relatively small, rendering the quality of the developed markers inadequate for comprehensive investigation of genetic diversity within *C. oleifera* populations.

With the rapid development of bioinformatics and continuous advancements in sequencing technology, the genome of *C. oleifera* has been successfully sequenced. Lin et al. [50] conducted transcriptome sequencing using the *C. oleifera* genome as a reference and detected 1,849,953 SNP molecular marker loci. Based on these SNP loci, they constructed high-quality molecular identities for 221 common *C. oleifera* resources. In this study, we performed ddRAD reduced-representation genome sequencing on 109 *C. oleifera* germplasms, using the *C. oleifera* genome as a reference and GATK4 for variant detection. A total of 6,252,197 population SNP loci and 722,237 population INDEL loci were identified. Compared to the study by Lin et al. [50], our study had a higher density of SNP molecular markers. Using ANNOVAR software for annotation analysis of the variant loci, we found that 145,679 SNPs and 12,756 INDELs were in exon regions. Variants in exon regions may lead to changes in the amino acids encoded by codons [51], and even mutations in start and stop codons, thereby altering the function of polypeptide chains and proteins and further affecting the traits of biological individuals. Therefore, variant loci located in exon regions have a significant impact on biological traits, and these variants may significantly influence the traits of *C. oleifera*.

Based on the combined results of Structure analysis, phylogenetic analysis, and principal component analysis, it is reasonable to divide the 109 *C. oleifera* germplasms into two groups: Q1 and Q2. The average Fst value across all SNP markers in the entire genome between these two groups is 0.0153, indicating a very weak level of differentiation between the two populations. Lin et al. [50] classified 221 *C. oleifera* materials into eight subgroups based on genetic structure, with Fst values ranging from 0 to 0.0085 among the subgroups. These findings further indicate a weak level of differentiation among the *C. oleifera* subgroups. Our results are consistent with their findings. Furthermore, Huang [52] used SSR molecular markers to analyze *C. oleifera* populations and found that the genetic differentiation coefficient of populations is lower than that of woody angiosperms. Therefore, the weak differentiation of *C. oleifera* populations may be attributed to the fact that it is a cross-pollinated plant that has undergone long-term domestication and extensive hybridization, leading to high gene flow among subgroups.

The average nucleotide diversity index for the Q1 subgroup is 4.45 × 10^−4^. These results are generally consistent with those of Chen et al. [48]. However, compared to Brazilian cassava plants (π=0.274) [53], the nucleotide diversity index of *C. oleifera* populations is very low, indicating a relatively low level of genetic diversity in germplasms. This may be due to the long-term domestication of *C. oleifera* [54]. To obtain high-yielding and high-quality *C. oleifera* varieties, plants have been subjected to long-term domestication, targeted breeding, and continuous selection, resulting in improved agronomic traits. However, long-term domestication has favored alleles that establish population advantages, while relatively disadvantageous alleles have been eliminated. Over time, this has led to a decrease in genetic diversity within *C. oleifera* populations [50]. The Tajima’s D values for both subgroups are greater than zero, indicating the presence of rare alleles at low frequencies and a relatively high number of intermediate-frequency alleles in both subgroups. This may be due to the similar growth environments of *C. oleifera*, leading to uniform selection pressure across the population.

The whole-genome association analysis (GWAS) is based on linkage disequilibrium and involves correlation analysis between target traits and variation sites to identify significant variation sites, thereby uncovering candidate genes for the target traits. GWAS has made significant achievements in the mapping of complex traits and the improvement of crop varieties in both plants and animals, such as rice [55], apple [56], and walnut [57]. However, due to the gaps in the sequencing and annotation of the *C. oleifera* genome, there have been few studies using GWAS to analyze *C. oleifera* traits.

The LD decay pattern observed in *C. oleifera* (r^2^ = 0.0979 at 64 kb, based on 2.8 million genome-wide SNPs) is comparable to other outcrossing woody perennials. For example, LD decayed to 0.1 within 50–100 kb in poplar [58] and within 40–80 kb in walnut [59]. This rapid decay reflects the high recombination rates and large effective population sizes characteristic of these species. Based on these observations, we selected a conservative ±50 kb candidate gene window, which is slightly narrower than the empirical LD half-decay distance of 64 kb. This approach ensures that genes in meaningful LD (r^2^ ≥ 0.1) with the significant SNPs are captured while minimizing false positives.

We identified 157 genetic loci significantly associated with fruit traits in *C. oleifera*. Specifically, there were 9 significant loci for single fruit weight, 2 for fruit diameter, 6 for fruit shape index, 40 for peel thickness, 69 for seed number per fruit, 5 for fresh seed weight, 23 for fresh seed yield rate, 1 for dry seed yield rate, and 2 for dry kernel yield rate. Notably, despite its high broad-sense heritability (*H*^2^ = 0.929), no significant loci were identified for fruit height in this study. Several factors may explain this absence of associations. First, fruit height may be controlled by many small-effect loci that did not reach the significance threshold given our sample size. Second, the relatively low repeatability of fruit height (ICC = 0.543) suggests moderate measurement variability, which may have reduced statistical power. Third, the genetic architecture of fruit height might involve rare variants that were not captured by our SNP panel after MAF filtering (>0.05). Finally, it is possible that the key genomic regions regulating fruit height are located in poorly covered regions of the ddRAD-seq data. This phenomenon—high heritability but few detected associations—has been observed in other plant GWAS studies and is often attributed to polygenic architecture, environmental interactions, and limited sample size [60]. Future studies with larger populations and higher-density markers may help identify loci associated with this trait.

We further screened 110 candidate genes based on the significant SNP loci. Among these, 46 candidate genes showed GO annotations. Among them, 6 candidate genes belonged to molecular function, 9 to cellular component, and 31 to biological process, involving various gene functions. Remarkably, 16 out of the 31 candidate genes related to biological processes were involved in reproduction, indicating their important roles in the development of *C. oleifera* fruits. In the Kyoto Encyclopedia of Genes and Genomes (KEGG) annotation, 23 genes were successfully annotated, primarily associated with genetic information-processing protein families, metabolic protein families, and terpenoid and polyketide metabolism. These significant SNP loci and candidate genes may be involved in multiple traits such as yield, fruit size, and peel thickness, providing important genetic information and molecular markers for the improvement of quality and yield of *C. oleifera*.

In our study, the annotation results of significant SNP sites indicate that most SNP markers are in intergenic regions. This may be attributed to the widespread distribution of intergenic regions in the *C. oleifera* genome [61]. Intergenic regions were once considered non-functional; however, with the completion of sequencing for an increasing number of plant genomes, it has been revealed that intergenic regions contain open chromatin. Moreover, it has been observed that larger plant genomes exhibit a higher proportion of open chromatin within their intergenic regions. Chromatin accessibility is involved in plant environmental responses, growth, and development [62,63].

Therefore, candidate regions—including both genic regions and intergenic regions harboring significant SNP sites—have the potential to influence corresponding traits. Intergenic regions may contain regulatory elements such as promoters, enhancers, and open chromatin regions that can influence the expression of nearby genes [64,65]. In plants with large genomes like *C. oleifera*, intergenic regions constitute a substantial proportion of the genome and may play important roles in environmental responses, growth, and development.

The functional implications of intergenic SNPs warrant further investigation. Future studies integrating RNA-Seq and comparative proteomic analysis of accessions with extreme phenotypes will be essential to validate whether these intergenic variants affect gene expression levels and subsequently contribute to phenotypic variation. Additionally, the non-synonymous SNPs identified in coding regions (90,876 SNPs; Figure 1) may have noticeable effects on protein sequences and functions.

Among the candidate genes identified in this study, several belong to functionally important gene families with known roles in plant development and stress responses. Cytochrome P450 genes (e.g., Chr08: 118268465 and Chr14: 35355883) are involved in the biosynthesis of secondary metabolites, hormones, and defense compounds, and have been implicated in fruit development and ripening in various plant species [66]. Protein kinases (e.g., Chr08: 96873006) play central roles in signal transduction pathways regulating cell division, differentiation, and responses to environmental stimuli [67]. Ubiquitin system components (e.g., Chr10: 63527409) are key regulators of protein degradation and have been shown to control fruit size and quality through the modulation of hormone signaling pathways [68,69]. These variants, particularly those in genes encoding cytochrome P450, protein kinases, and ubiquitin system components (Table 1), represent promising candidates for functional validation through gene editing or heterologous expression systems in future *C. oleifera* research.

This study offers novel insights and empirical data to support research on the genetic evolution and genomic structure of *C. oleifera*. Moving forward, the integration of significant SNP loci and candidate genes will facilitate more comprehensive functional studies and gene editing experiments, thereby elucidating the molecular mechanisms underlying key traits of *C. oleifera*. This will provide more reliable and effective technical support for the genetic breeding and industrial development of *C. oleifera*.

## 5. Conclusions

This study performs a genetic diversity analysis of 109 *C. oleifera* germplasm accessions using fruit phenotypic traits and SNP molecular markers. GWAS identified significant SNP loci and candidate genes influencing fruit phenotypic traits. By integrating structure analysis, phylogenetic analysis, and PCA, we classified the 109 *C. oleifera* germplasm accessions into two distinct populations. The genetic differentiation between the two subgroups was weak, with nucleotide diversity indices (π) of 4.45 × 10^−4^ and 5.35 × 10^−4^, respectively, indicating low genetic diversity in *C. oleifera* germplasms. This may be attributed to long-term domestication of the species. The Tajima’s D values for both subpopulations were greater than zero, suggesting the presence of numerous intermediate-frequency alleles. This may be attributed to the similar environments in which the *C. oleifera* germplasm grows, leading to uniform selective pressures within the population. GWAS was employed to pinpoint loci significantly associated with fruit traits in the germplasm resources. The proteins encoded by the identified candidate genes were primarily involved in various biological processes, including biosynthesis, regulation of stem system morphogenesis, reproduction, plant organ development, transport, and metabolic pathways. These proteins include plant hormone glycosyltransferases, peptidases and inhibitors, ubiquitin protein ligases, cytochrome P450s, and protein kinases. Notably, among the candidate genes related to 31 biological processes, 16 were specifically involved in reproduction. This highlights the crucial role these candidate genes play in the development of *C. oleifera* fruits. Our findings provide novel insights and data support for the study of genetic evolution and genomic structure in *C. oleifera*. Furthermore, they hold significant importance for the targeted breeding of elite varieties, paving the way for future improvements in *C. oleifera* cultivation.

## Figures and Tables

**Table 1:** KEGG pathway enrichment for candidate genes.

Trait	SNP	Gene ID	KEGG Pathway
Fruit shape index	Chr02: 20212286	maker-Chr2-snap-gene-202.6-mRNA-1	Messenger RNA biogenesis
Number of seeds per fruit	Chr01: 84240256	augustus_masked-Chr1-processed-gene-841.29-mRNA-1	Transcription factors
	Chr03: 105301961	augustus_masked-Chr3-processed-gene-1053.14-mRNA-1	Ribosome
	Chr03: 167365123	maker-Chr3-snap-gene-1673.21-mRNA-1	Ribosome biogenesis
	Chr08: 96873006	maker-Chr8-snap-gene-969.6-mRNA-1	Protein kinases
	Chr08: 114566238	maker-Chr8-snap-gene-1146.2-mRNA-1	Carotenoid biosynthesis
	Chr08: 118268465	genemark-Chr8-processed-gene-1182.39-mRNA-1	Cytochrome P450
	Chr10: 63527409	maker-Chr10-snap-gene-635.3-mRNA-1	Ubiquitin system
	Chr10: 134255167	maker-Chr10-snap-gene-1342.19-mRNA-1	Enzymes with EC numbers
	Chr11: 153463775	snap_masked-Chr11-processed-gene-1534.13-mRNA-1	Chromosome and associated proteins
	Chr14: 35355883	augustus_masked-Chr14-processed-gene-353.13-mRNA-1	Cytochrome P450
	Chr14: 114275276	snap_masked-Chr14-processed-gene-1142.45-mRNA-1	Transcription factors
Peel thickness	Chr01: 6227267	maker-Chr1-snap-gene-62.12-mRNA-1	Cysteine and methionine metabolism
	Chr04: 159617039	maker-Chr4-snap-gene-1596.0-mRNA-2	Membrane trafficking
	Chr05: 167572639	maker-Chr5-snap-gene-1676.3-mRNA-1	Exosome
	Chr07: 41713895	maker-Chr7-snap-gene-417.23-mRNA-1	Transcription factors
	Chr09: 72920012	maker-Chr9-snap-gene-729.27-mRNA-1	Protein kinases
	Chr13: 135481819	augustus_masked-Chr13-processed-gene-1354.42-mRNA-1	Membrane trafficking
Fruit height	Chr07: 49799009	maker-Chr7-snap-gene-497.36-mRNA-2	Peptidases and inhibitors
	Chr09: 85815245	maker-Chr9-snap-gene-858.43-mRNA-1	Ubiquitin system
	Chr14: 97790223	maker-Chr14-snap-gene-977.10-mRNA-1	Glycosyltransferases
Fresh seed yield	Chr02: 158899204	maker-Chr2-snap-gene-1588.1-mRNA-1	Chaperones and folding catalysts
	Chr08: 106777293	augustus_masked-Chr8-processed-gene-1068.0-mRNA-1	Transcription factors

**Table 2:** GO functional annotation of candidate genes.

Candidate Gene	GO Category	Annotation
maker-Chr4-snap-gene-1582.40-mRNA-1	molecular_function	function unknown
maker-Chr7-snap-gene-82.12-mRNA-1	molecular_function	heterocyclic compound binding
maker-Chr12-snap-gene-379.34-mRNA-1	molecular_function	nucleotide binding
maker-Chr14-snap-gene-1071.26-mRNA-1	molecular_function	nucleotide binding
augustus_masked-Chr10-processed-gene-2066.42-mRNA-1	molecular_function	heterocyclic compound binding
augustus_masked-Chr11-processed-gene-1405.6-mRNA-1	molecular_function	function unknown
maker-Chr2-snap-gene-1558.2-mRNA-1	cellular_component	bounding membrane of organelle
augustus_masked-Chr3-processed-gene-1053.14-mRNA-1	cellular_component	protein-containing complex
maker-Chr3-snap-gene-1673.21-mRNA-1	cellular_component	protein-containing complex
maker-Chr8-snap-gene-1008.6-mRNA-2	cellular_component	bounding membrane of organelle
genemark-Chr8-processed-gene-1182.39-mRNA-1	cellular_component	endoplasmic reticulum subcompartment
augustus_masked-Chr10-processed-gene-634.18-mRNA-1	cellular_component	bounding membrane of organelle
maker-Chr10-snap-gene-635.3-mRNA-1	cellular_component	ubiquitin ligase complex
maker-Chr10-snap-gene-1524.3-mRNA-1	cellular_component	endoplasmic reticulum subcompartment
maker-Chr2-snap-gene-1588.1-mRNA-1	cellular_component	bounding membrane of organelle
maker-Chr2-snap-gene-202.6-mRNA-1	biological_process	reproduction
augustus_masked-Chr2-processed-gene-593.9-mRNA-1	biological_process	RNA biosynthetic process
maker-Chr5-snap-gene-268.33-mRNA-1	biological_process	RNA biosynthetic process
augustus_masked-Chr1-processed-gene-841.29-mRNA-1	biological_process	reproduction
maker-Chr4-snap-gene-1150.47-mRNA-1	biological_process	reproduction
maker-Chr5-snap-gene-1262.30-mRNA-1	biological_process	reproduction
snap_masked-Chr6-processed-gene-475.2-mRNA-1	biological_process	defense response to other organism
augustus_masked-Chr6-processed-gene-670.4-mRNA-1	biological_process	reproduction
maker-Chr8-snap-gene-969.6-mRNA-1	biological_process	cellular response to hormone stimulus
maker-Chr8-snap-gene-1146.2-mRNA-1	biological_process	monocarboxylic acid metabolic process
maker-Chr10-snap-gene-1342.19-mRNA-1	biological_process	transition metal ion transport
maker-Chr11-snap-gene-1345.57-mRNA-1	biological_process	RNA biosynthetic process
augustus_masked-Chr12-processed-gene-1709.3-mRNA-1	biological_process	reproduction
augustus_masked-Chr14-processed-gene-353.13-mRNA-1	biological_process	monocarboxylic acid metabolic process
snap_masked-Chr14-processed-gene-1142.45-mRNA-1	biological_process	reproduction
maker-Chr15-snap-gene-1364.34-mRNA-1	biological_process	reproduction
maker-Chr1-snap-gene-62.12-mRNA-1	biological_process	reproduction
maker-Chr4-snap-gene-1596.0-mRNA-2	biological_process	autophagosome assembly
maker-Chr5-snap-gene-1275.2-mRNA-1	biological_process	reproduction
maker-Chr5-snap-gene-1676.3-mRNA-1	biological_process	transition metal ion transport
maker-Chr7-snap-gene-376.6-mRNA-1	biological_process	regulation of shoot system morphogenesis
maker-Chr7-snap-gene-417.23-mRNA-1	biological_process	reproduction
maker-Chr9-snap-gene-729.27-mRNA-1	biological_process	reproduction
maker-Chr9-snap-gene-1211.11-mRNA-1	biological_process	autophagosome assembly
augustus_masked-Chr13-processed-gene-1354.42-mRNA-1	biological_process	regulation of localization
maker-Chr5-snap-gene-439.0-mRNA-1	biological_process	reproduction
maker-Chr7-snap-gene-497.36-mRNA-2	biological_process	monocarboxylic acid metabolic process
maker-Chr11-snap-gene-848.23-mRNA-1	biological_process	reproduction
maker-Chr14-snap-gene-977.10-mRNA-1	biological_process	reproduction
augustus_masked-Chr11-processed-gene-881.28-mRNA-1	biological_process	plant organ development
maker-Chr5-snap-gene-439.0-mRNA-1	biological_process	reproduction

## Data Availability

All data and materials are available upon request from the corresponding authors.

## Supplementary Materials

The following supporting information can be downloaded at: https://www.mdpi.com/article/10.3390/biology15060483/s1, File S1: The information of 109 *Camellia oleifera* germplasm accessions; File S2: Phenotypic data of the ten fruit traits for the 109 *Camellia oleifera* germplasm accessions; File S3: Summary of significant loci identified by GWAS for ten fruit traits in *Camellia oleifera*; File S4: ICC and *H*^2^ estimates for ten fruit traits in *Camellia oleifera*; File S5: *Camellia oleifera* germplasm sequencing results; Supplementary Figure S1: correlation; Supplementary Figure S2: LD_decay.
