# Supplementary figures and images for: Genome-Wide Association Study of Fruit Traits Using 109 Germplasm Accessions of Camellia oleifera
Source: Biology (Basel). 2026 Mar 18;15(6):483. doi: 10.3390/biology15060483 (PMC13024145; doi:10.3390/biology15060483)

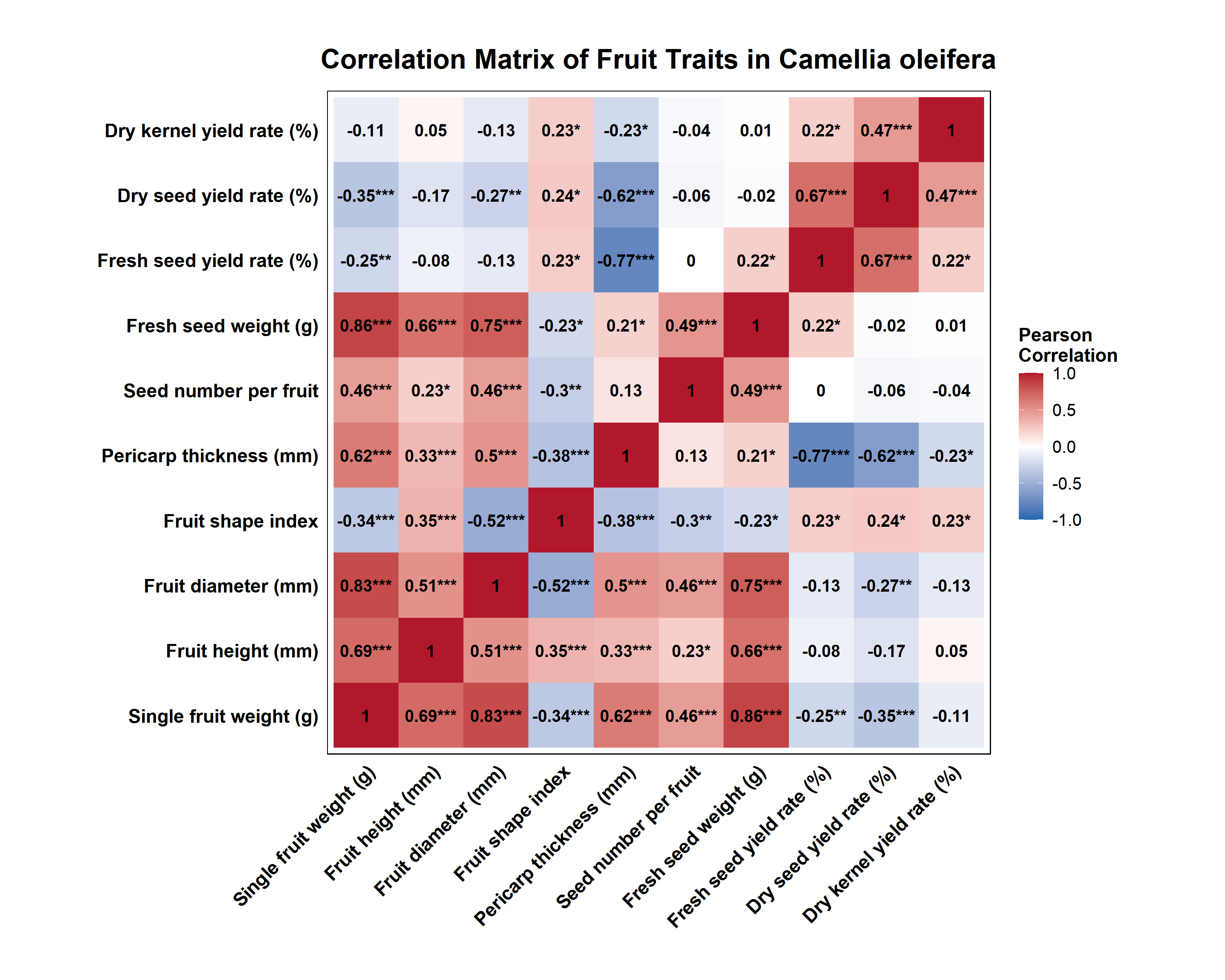

Supplement: Supplementary file 1 [file biology-15-00483-s001.zip › Figure_S1_correlation.tiff]

**Figure S2. *Camellia oleifera* LD decay**

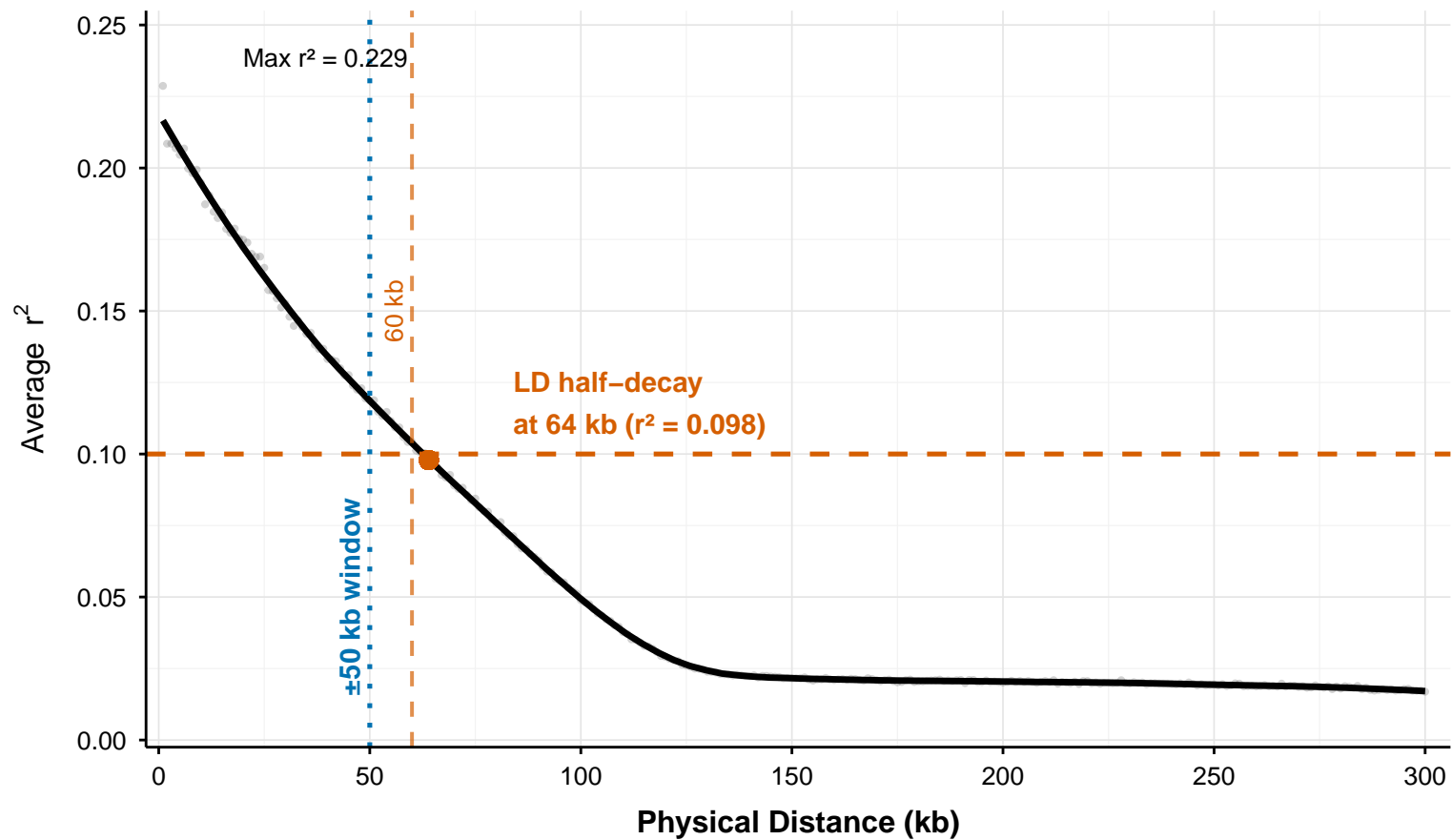

Supplement: Supplementary file 1 [file biology-15-00483-s001.zip › Figure_S2_LD_decay.pdf]
